# Structural performance of the concrete-filled tube column with internal triangular units subjected to axial compression

**DOI:** 10.1371/journal.pone.0297154

**Published:** 2024-03-06

**Authors:** Jinwon Kim, Se-Jung Lee, Sang-Hyun Ji, Dae-Jin Kim

**Affiliations:** 1 Steel Solution Center, POSCO, Incheon, Republic of Korea; 2 SEJIN Research and Service Co., Seoul, Republic of Korea; 3 Department of Architectural Engineering, Kyung Hee University, Yongin, Gyeonggi, Republic of Korea; Universiti Teknologi Malaysia, MALAYSIA

## Abstract

This study introduces a novel concrete-filled tube (CFT) column system featuring a steel tube comprised of four internal triangular units. The incorporation of these internal triangular units serves to reduce the width-thickness ratio of the steel tube and augment the effective confinement area of the infilled concrete. This design enhancement is anticipated to result in improved structural strength and ductility, contributing to enhanced overall performance and sustainability. To assess the effectiveness of the newly proposed column system, a full-scale test was conducted on five square steel tube column specimens subjected to axial compression. Among these specimens, two adhered to the conventional steel tube column design, while the remaining three featured the new CFT columns with internal triangular units. The shape of the CFT column, the presence of infilled concrete and the presence of openings on the ITUs were considered as test parameters. The test results reveal that the ductility of the newly proposed CFT column system exhibited a minimum 30% improvement compared to the conventional CFT column. In addition, the initial stiffness and axial compressive strength of the new system were found to be comparable to those of the conventional CFT column.

## 1. Introduction

The concrete-filled tube (CFT) column is a composite structural member, which is composed of the rectangular (or circular) steel tube and concrete filled inside the tube [1–5]. This combination is highly effective because the presence of the steel tube enhances the confinement effect of the infilled concrete. The steel tube is capable of resisting bending moments, while the infilled concrete can withstand compression forces acting on the column [6–11]. Furthermore, the infilled concrete is helpful to mitigate the local buckling and torsional deformation of the steel tube. Consequently, the CFT is a very efficient structural system in terms of both strength and ductility.

The integration of the steel tube and infilled concrete is crucial for ensuring safe load transfer from the upper floor to the lower one in CFT columns. This integration is primarily achieved through friction at the interface of the two components. However, in cases where the frictional resistance is insufficient, additional shear connectors must be affixed to the steel tube. Unfortunately, this supplementary measure can negatively impact the ease of construction and the cost-effectiveness of CFT columns [12–17].

Another important issue considered in the design of the CFT column is the limitation on the width-thickness ratio of the steel tube. Utilizing an exceptionally thin steel tube for CFT columns subjected to high compression forces with relatively low bending moments can offer economic advantages. Nevertheless, it may also lead to local buckling of the steel tube, resulting in a diminished confinement effect on the infilled concrete [18–23]. Due to this potential concern, major steel design codes such as the American Institute of Steel Construction (AISC) Load and Resistance Factor Design (LRFD) [24] and Eurocode 4 [25] impose limitations on the width-thickness ratio of CFT columns, as indicated in Table 1. This table outlines the permissible range of concrete compressive strength, steel yield strength and the maximum allowable width-thickness ratio for both rectangular and circular steel tubes.

**Table 1:** Design code provisions for CFT columns.

Design code	AISC LRFD		Eurocode 4	
*A*_*s*_ */A*_*g*_	1% or above		2 ~ 9%	
$f_{c}\prime$ (MPa)	21 ~ 70		20 ~ 50	
$F_{y}^{\text{max}}$ (MPa)	440		450	
Shape	□	○	□	○
*B/t*	$\sqrt{\frac{5.1E}{F_{y}}}$	$\frac{0.15E}{F_{y}}$	$52\sqrt{\frac{235}{F_{y}}}$	$90\frac{235}{F_{y}}$

**Fig 1:**
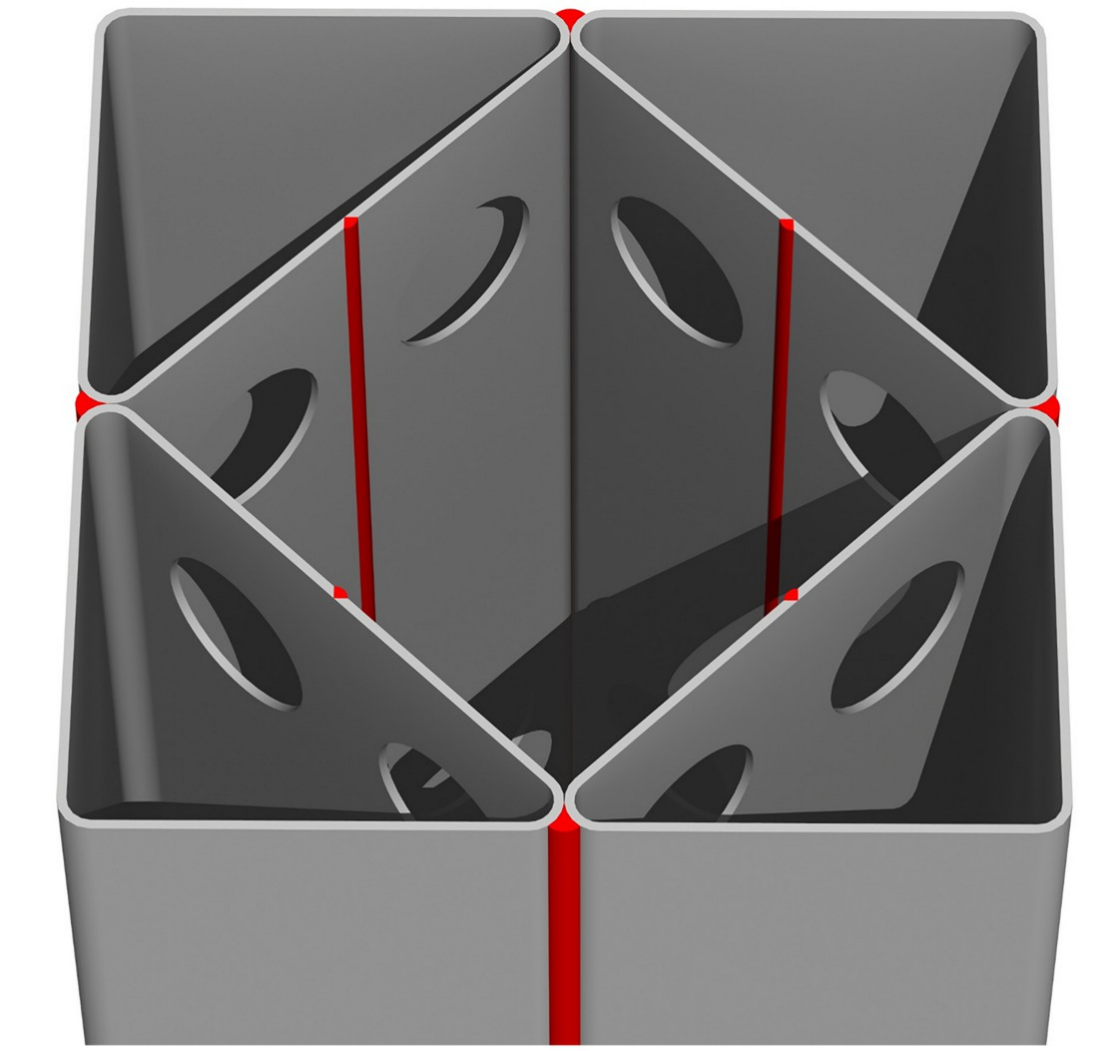
Exterior view of the newly proposed CFT-ITU column.

**Fig 2:**
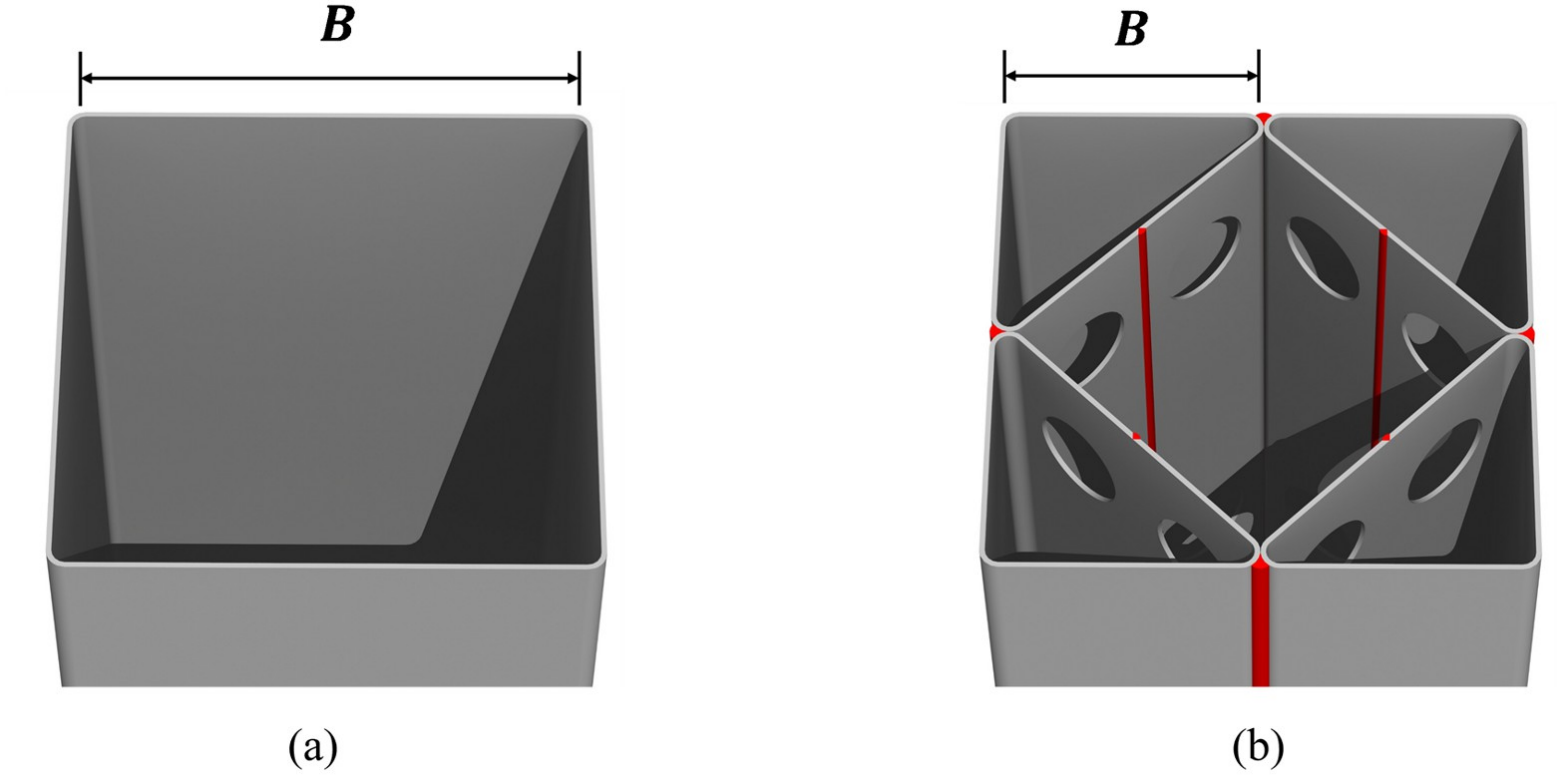
Determination of the width-thickness ratio. (a) Conventional CFT column. (b) Newly proposed CFT-ITU column.

Considering all the issues discussed above, we propose a new CFT column (CFT-ITU), which consists of four steel internal triangular units (ITUs), as shown in Fig 1. In this system, the presence of the ITUs can basically increase the contact area between the steel tube and infilled concrete, and thus the integration of the two components can be improved. As several circular holes exist on the internal face of each triangular unit, they function as perfobond shear connectors [26–30] and can guarantee an excellent composite behavior between the steel tube and infilled concrete without attaching additional shear connectors. In addition, the use of the four internal triangular units allows the reduction of the width-thickness ratio of the steel tube by half as illustrated in Fig 2. This can clearly increase the local buckling resistance capacity of the rectangular steel tube.

**Fig 3:**
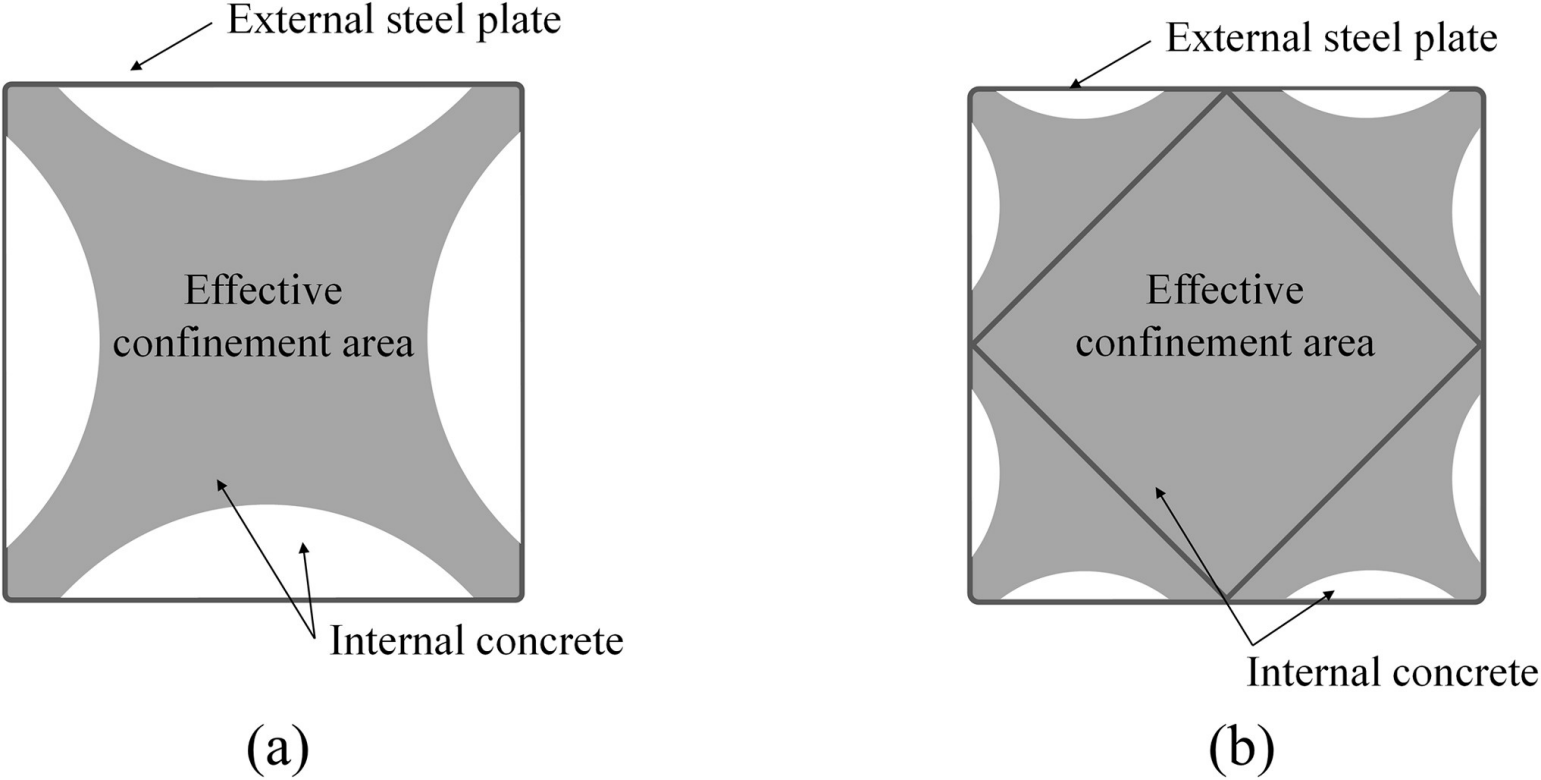
Effective confinement area. (a) Conventional CFT column. (b) Newly proposed CFT-ITU column.

One of the main advantages of the CFT column is the confinement effect of the infilled concrete due to the presence of the steel tube [31–37]. When a compressive force is applied to the CFT column, the infilled concrete experiences shrinkage in the loading direction and expansion in the direction perpendicular to the applied load, as per the well-known Poisson’s effect. This expansion is restricted by the external steel tube, thereby enhancing the compressive strength of the infilled concrete. However, it is worth noting that this advantageous confinement effect is often not considered in the design of rectangular CFT columns. This is primarily because it is most noticeable at the corners of the rectangular column, rather than around the central region of each side of the rectangular cross-section, as illustrated in Fig 3(a).

**Fig 4:**
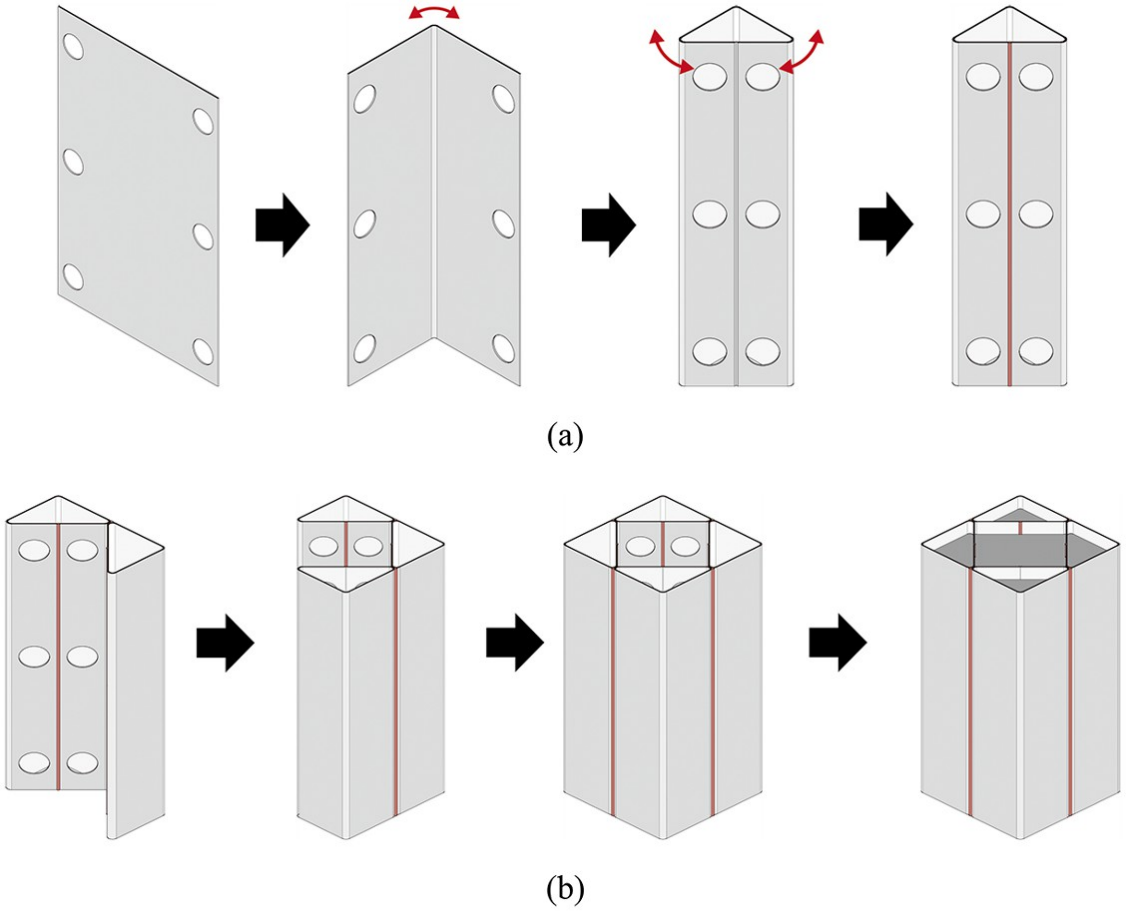
Construction process of the newly proposed CFT-ITU column. (a) Internal triangular unit. (b) CFT-ITU column with infilled concrete.

In contrast, the presence of the internal triangular units can increase the effective confinement area of the internal concrete in the proposed CFT-ITU column system, as illustrated in Fig 3(b). This feature can apparently compensate for the disadvantage of the existing rectangular CFT column with regard to the confinement effect. Fig 4 shows the construction process of the proposed CFT-ITU column system. In this process, an internal triangular unit can be created by folding a rectangular steel plate with three punched holes and subsequently welding its edges. Afterward, the four internal triangular units are welded together along the sections highlighted by red lines in the figure. Once this assembly is complete, concrete is poured into the steel tube structure.

In this paper, a full-scale test was conducted on five square steel tube column specimens subjected to axial load to assess the effectiveness of the proposed system. Two of these specimens were conventional steel tube columns, while the other three were newly proposed CFT-ITU columns. Test parameters included the presence of infilled concrete and holes on the internal triangular units. The failure characteristics of the test specimens were examined, and their load-displacement curves were analyzed. Furthermore, we investigated the effects of the test parameters on the peak strength and ductility of the test specimens in order to demonstrate the effectiveness of the proposed system.

## 2. Experimental program

### 2.1 Test specimens

**Fig 5:**
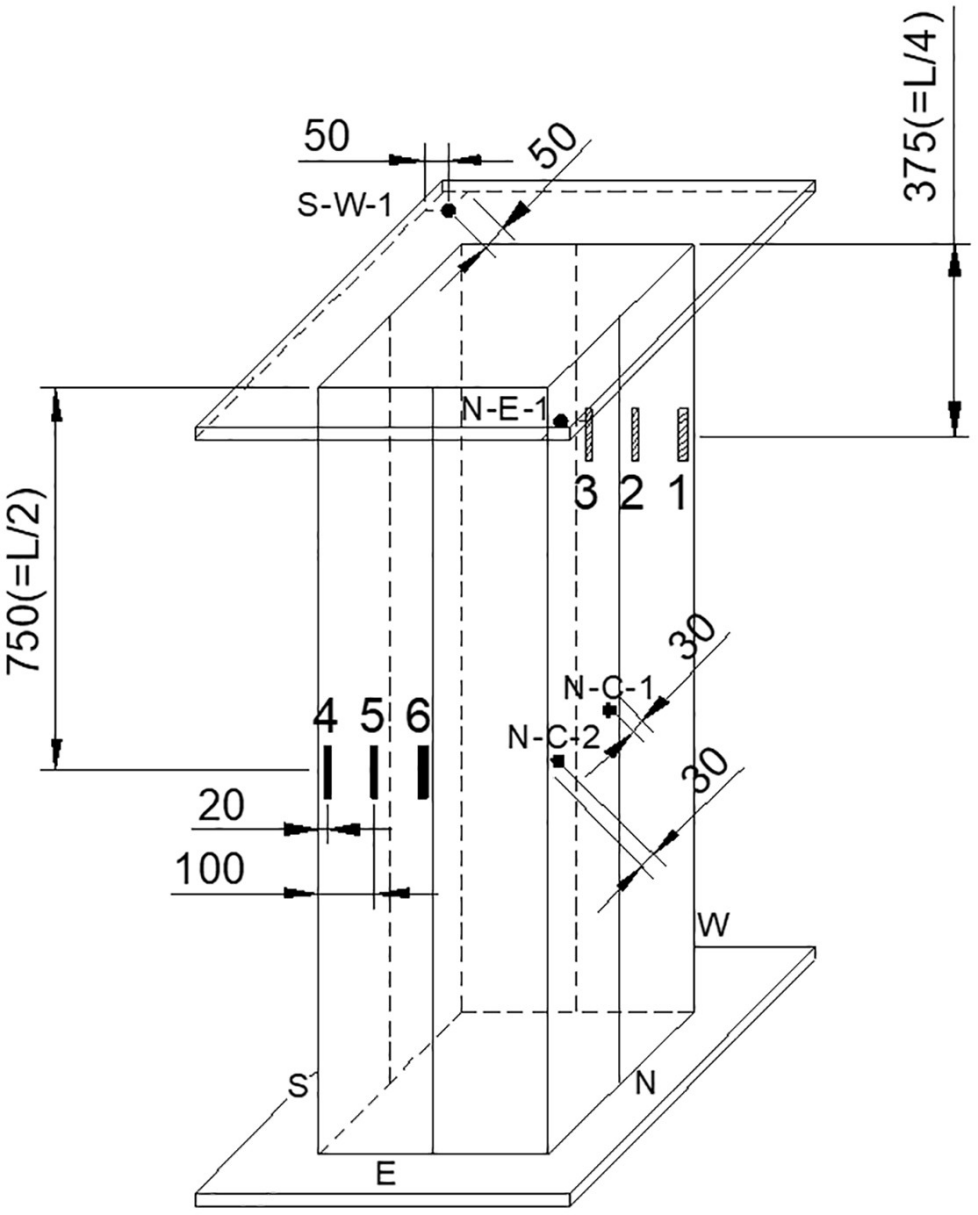
Dimensions of the test specimen.

For this study, a total of five square steel tube column specimens were manufactured and tested to investigate the effectiveness of the newly proposed CFT-ITU column system. Two of them were the conventional steel tube columns, and the other three specimens the newly proposed CFT-ITU columns. The dimension of all the column specimens is the same as 400 mm × 400 mm (cross-sectional dimension) × 1500 mm (height). The steel tube thickness of the two conventional steel tube specimens is 7 mm, while that of the other three ITU specimens 4 mm. Fig 5 illustrates the geometrical configurations of the specimens described above. Steel plates of 40 mm thickness were attached to both ends of the test specimen in order to evenly distribute the applied compressive force and prevent local buckling from occurring.

The details of the five test specimens are outlined in Table 2, with the main test parameters encompassing the shape of the concrete-filled tube (CFT) column, the presence of infilled concrete, and the existence of openings on the internal triangular units (ITUs). The specimens are categorized into two types of CFT column systems: the conventional steel tube specimens (ST) and the newly proposed CFT-ITU specimens (ITU). The two ST specimens consist of one with infilled concrete (C24) and one without infilled concrete (NC). Similarly, the three ITU specimens include two without infilled concrete (NC) and one with infilled concrete (C24). Among the ITU specimens without infilled concrete, one has openings on the ITUs (O), while the other does not (NO). The compressive strength of concrete (*f’*_*c*_) was measured in accordance with the ASTM C39/C39M-11 standard [38], and the measured average strength was found to be 30.0 MPa. The yield strength of the steel plate (*F*_*y*_) used in each specimen was measured per the ASTM A370-12a standard [39].

**Table 2:** Details of test specimens.

No.	Specimen	Column size (mm)	Material strength (MPa)		Presence of openings on ITUs
			Steel	Concrete	
1	ST_7t_NC_NO	400×400×7t	454.0	N/A	No
2	ST_7t_C24_NO			30.0	
3	ITU_4t_NC_NO	400×400×4t	420.0	N/A	
4	ITU_4t_NC_O				Yes
5	ITU_4t_C24_O			30.0	

**Fig 6:**
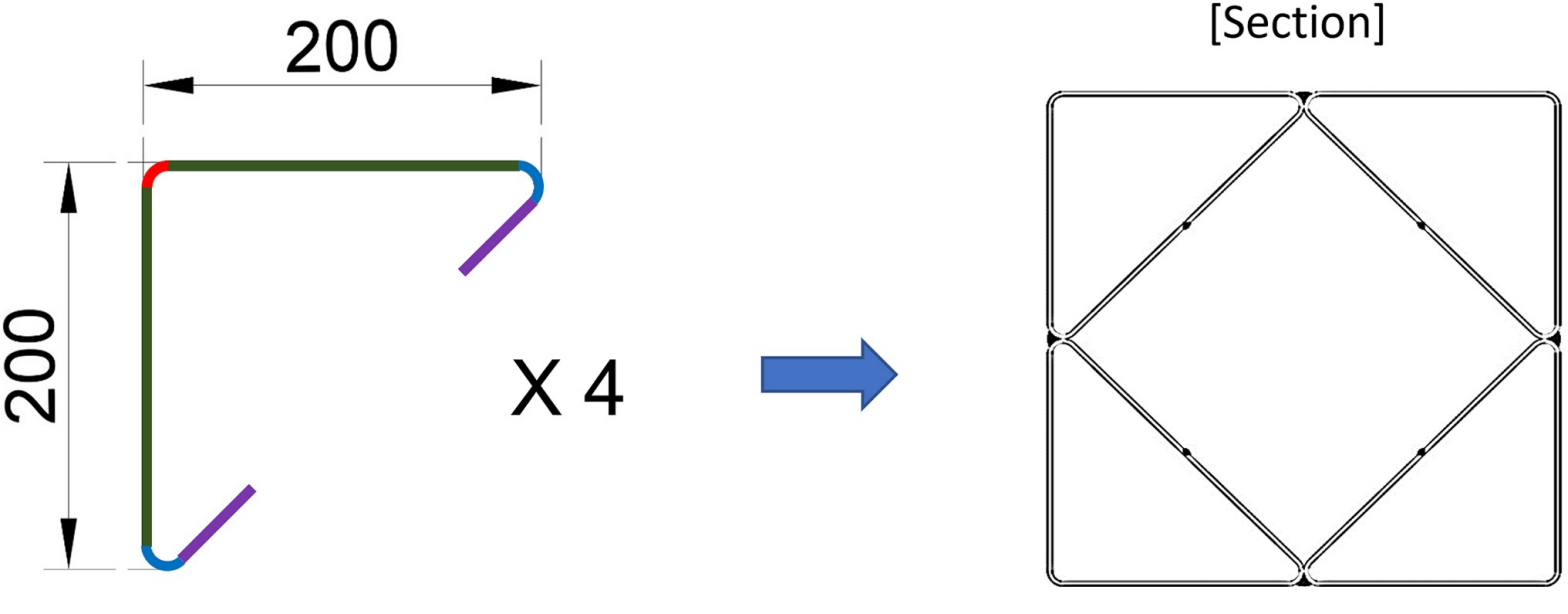
Steel column tube consisting of four internal triangular units.

**Fig 7:**
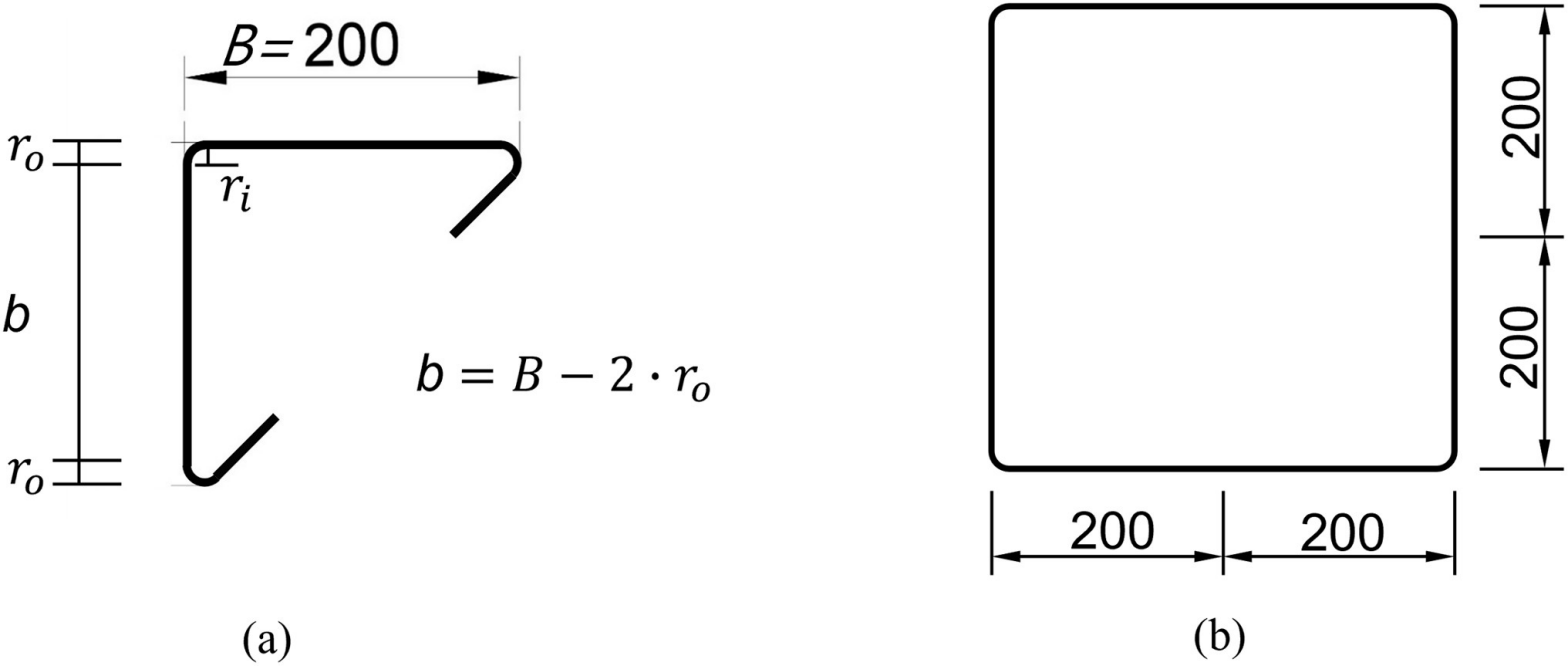
Cross-sectional area of the steel tube. (a) ITU specimen. (b) Conventional CFT specimen.


Fig 6 shows the cross-sectional shape of the steel tube for the ITU specimens. In Fig 7, some detailed dimensions of the ITU and conventional steel tube specimens are provided. The cross-sectional areas of the steel and concrete components are required to estimate the nominal compressive strength of each specimen based on the AISC LRFD specification, as discussed in Section 3. The cross-sectional areas of the steel tube (*A*_*s*_) for ITU specimens with and without holes on ITUs, and conventional steel tube specimens can be calculated using Eqs (1) to (3), respectively, as given below.

$$A_{s1}=4t\left[\left(2+\sqrt{2}\right)\left(B-2r_{o}\right)+\pi \left(r_{o}+r_{i}\right)-R\right],\left(\text{ITU}\;\text{w}/\text{holes}\;\text{on}\;\text{ITUs}\right)$$ (1)

$$A_{s2}=4t\left[\left(2+\sqrt{2}\right)\left(B-2r_{o}\right)+\pi \left(r_{o}+r_{i}\right)\right],\left(\text{ITU}\;\text{w}/\text{o}\;\text{holes}\;\text{on}\;\text{ITUs}\right)$$ (2)

$$A_{s3}=4t\left(B-t\right)-\left(4-\pi \right)\left(r_{o}^{2}-r_{i}^{2}\right).\left(\text{Conventional}\;\text{steel}\;\text{tube}\right)$$ (3)

Here, *B* and *t* are the size of the square cross-section and thickness of the steel tube, respectively. *r*_*o*_ and *r*_*i*_ are the outer and inner radii of the steel tube corner, respectively, as indicated in Fig 7(a). *R* is the radius of the holes on the ITUs. The calculation of the cross-sectional area of steel components using Eq (1) is not precise due to the presence of holes at specific levels of the internal triangular units. This discrepancy may lead to an underestimation of the nominal compressive strengths of the ITU specimens, but it is a conservative approach.

The gross area of all column specimens (*A*_*g*_) can be computed using Eq (4), and the cross-sectional area of infilled concrete (*A*_*c*_) can be calculated by plugging one of the three equations from (1) to (3) into *A*_*s*_ of Eq (5).


$$A_{g}=B^{2}-\left(4-\pi \right)r_{o}^{2},$$ (4)

$$A_{c}=A_{g}-A_{s}.$$ (5)

The cross-sectional areas of each test specimen are summarized in Table 3. The values on the table were obtained using Eqs (1) to (5) provided above.

**Table 3:** Cross-sectional areas of each test specimen.

No.	Specimen	*t* (mm)	*r*_*o*_ (mm)	*r*_*i*_ (mm)	*A*_*g*_ (mm^2^)	*A*_*s*_ (mm^2^)	*A*_*c*_ (mm^2^)
1	ST_7t_NC_NO	7.0	24.5	17.5	N/A	10,752	N/A
2	ST_7t_C24_NO				159,485		148,733
3	ITU_4t_NC_NO	4.0	14.0	10.0	N/A	10,602	N/A
4	ITU_4t_NC_O					8,202	
5	ITU_4t_C24_O				159,832		151,630

*t* = thickness of steel tube, *r*_*o*_ (*r*_*i*_) = outer (inner) radius of steel tube corner, *A*_*g*_ = gross area of column specimen, *A*_*s*_ = cross-sectional area of steel tube, *A*_*c*_ = cross-sectional area of infilled concrete.

### 2.2 Testing equipment and procedure

**Fig 8:**
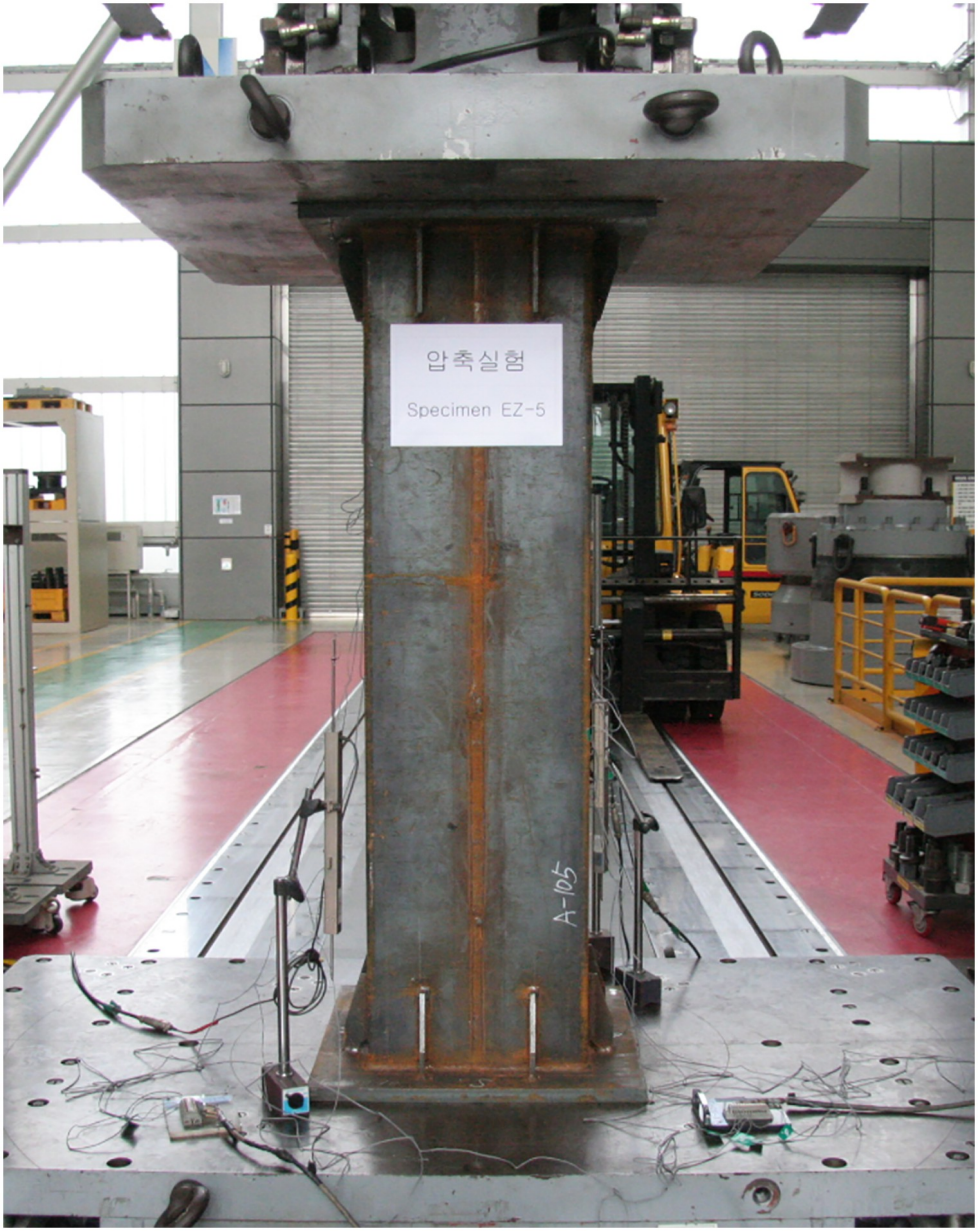
Test setup.

The test setup is illustrated in Fig 8. The test was carried out using a universal testing machine (UTM) equipped with displacement-based load control capabilities. The UTM has a maximum capacity of 10,000 kN, and the load was applied to the specimen at a rate of 0.01 mm/s. Two linear variable differential transducers (LVDTs) were installed to measure the deformation of the specimen at various locations as shown in the figure. Their average was used to plot the load-displacement curve of each specimen. The load-versus-displacement data were recorded throughout the entire loading history using a computer-aided data acquisition system.

## 3. Strength estimation by AISC LRFD

This section discusses the AISC LRFD specification [24] to estimate the nominal compressive strengths of the five test specimens. This specification also provides insights on the potential failure mode of each specimen.

### 3.1 Compressive strengths of the steel tube columns

The rectangular steel tube column sections without any infilled concrete can be classified into two types such as slender and nonslender. If a shape is slender, its strength limit state is local buckling, and the corresponding reduced strength must be computed, according to AISC B4.1. The slenderness limit for the stiffened element at its both ends under uniform compression is given as $\lambda_{r}=1.49\sqrt{E/F_{y}}$. If the width-thickness ratio of the hollow shape *λ* (= *b*/*t*) is greater than this value, the nominal compressive strength must be reduced by applying the so-called reduction factor *Q* that can be computed using the following equations.


$$b_{e}=1.92t\sqrt{\frac{E}{F_{y}}}\left[1-\frac{0.38}{b/t}\sqrt{\frac{E}{F_{y}}}\right]\leq b,$$ (6)

$$Q=\frac{A_{eff}}{A_{s}}.$$ (7)

The nominal compressive strengths (*P*_*n*_) of the steel tube columns without infilled concrete can be computed using the critical buckling strength of the test specimens (*F*_*cr*_) and the cross-sectional areas of the steel tube (*A*_*s*_) computed by Eqs (1) to (3).


$$P_{n}=F_{cr}A_{s}.$$ (8)

The critical buckling strength *F*_*cr*_ can be determined depending on the slenderness ratio *KL*/*r*, as given below.

If $\frac{KL}{r}\leq 4.71\sqrt{\frac{E}{QF_{y}}}$ (inelastic buckling),

$$F_{cr}=Q\left(0.658^{\frac{QF_{y}}{F_{e}}}\right)F_{y}.$$ (9)

If $\frac{KL}{r}>4.71\sqrt{\frac{E}{QF_{y}}}$ (elastic buckling),

$$F_{cr}=0.877F_{e}.$$ (10)

Here, *F*_*e*_ is the elastic buckling strength stated by

$$F_{e}=\frac{\pi^{2}E}{{\left(KL/r\right)}^{2}}.$$ (11)

Using the above equations, the nominal compressive strengths of the three non-infilled specimens such as ST_7t_NC_NO, ITU_4t_NC_NO and ITU_4t_NC_O can be estimated as given in Tables 4 and 5.

**Table 4:** Reduction factor computation for the three non-infilled specimens.

Specimen	*λ*	*λ*_*r*_	*Q*	Section type
ST_7t_NC_NO	50.1	31.7	0.61	Slender
ITU_4t_NC_NO	43.0	32.9	0.70	
ITU_4t_NC_O				

*λ* = width-thickness ratio of steel tube, $\lambda_{r}=1.49\sqrt{E/F_{y}}$, *Q* = reduction factor.

**Table 5:** Nominal compressive strengths of the three non-infilled specimens.

Specimen	* $\frac{KL}{r}$ *	* $4.71\sqrt{\frac{E}{QF_{y}}}$ *	*F*_*cr*_ (MPa)	*P*_*n*_ (kN)	Global buckling type
ST_7t_NC_NO	4.7	128.0	277.3	2,981.8	Inelastic
ITU_4t_NC_NO	5.3	124.3	293.9	3,115.5	
ITU_4t_NC_O	4.9	124.3	293.9	2,410.5	

*KL*/*r* = slenderness ratio, *F*_*cr*_ = critical buckling strength, *P*_*n*_ = nominal compressive strength.

As shown in Table 4, the width-thickness ratio (*λ*) is smaller than the slenderness limit (*λ*_*r*_) in all three non-infilled specimens, and thus local buckling occurs. Consequently, their critical buckling strengths (*F*_*cr*_) must be reduced using *Q* factor. Furthermore, the slenderness ratios (*KL*/*r*) provided in Table 5 suggest that the primary failure mode for the three non-infilled specimens is inelastic buckling, as described by Eq (9).

### 3.2 Compressive strengths of the CFT columns

The nominal compressive strengths (*P*_*n*_) of the rectangular CFT columns with infilled concrete can be computed depending on the ratio of *P*_*no*_ to *P*_*e*_, as given below. Here, *P*_*no*_ is the nominal compressive strength of the infilled CFT columns, in which length effects are not considered, and *P*_*e*_ the elastic buckling strength.

If *P*_*no*_/*P*_*e*_ ≤ 2.25,

$$P_{n}=P_{no}\left[0.658^{\left(P_{no}/P_{e}\right)}\right].$$ (12)

If *P*_*no*_/*P*_*e*_ > 2.25,

$$P_{n}=0.877P_{e}.$$ (13)

The elastic buckling strength *P*_*e*_ can be determined using the following equations.


$$P_{e}=\frac{\pi^{2}{\left(EI\right)}_{eff}}{{\left(KL\right)}^{2}},$$ (14)

$${\left(EI\right)}_{eff}=E_{s}I_{s}+C_{3}E_{c}I_{c},$$ (15)

$$C_{3}=0.6+2\left(\frac{A_{s}}{A_{c}+A_{s}}\right)\leq 0.9.$$ (16)

According to AISC I2.2, the compressive strength of concrete-filled hollow sections is the limit state of flexural buckling, of which strength can be reduced by considering the effects of local buckling. With this background, the determination of *P*_*no*_ depends on the width-thickness ratio of the hollow CFT section λ (= *b*/*t*), which can be categorized into three types such as compact, noncompact and slender. (Refer to Fig 7 to compute the width-thickness ratio λ.) The two critical values of the width-thickness ratio are given by $\lambda_{p}=2.26\sqrt{E/F_{y}}$ and $\lambda_{r}=3.00\sqrt{E/F_{y}}$, and the width-thickness ratio λ must not be greater than $5.00\sqrt{E/F_{y}}$.

If *λ*≤*λ*_*p*_ (compact section),

$$P_{no}=P_{p},$$ (17)

in which

$$P_{p}=F_{y}A_{s}+0.85f_{c}^{\prime }A_{c}.$$ (18)

If *λ*_*p*_ < *λ* ≤ *λ*_*r*_ (noncompact section),

$$P_{no}=P_{p}-\frac{P_{p}-P_{y}}{{\left(\lambda_{r}-\lambda_{p}\right)}^{2}}{\left(\lambda -\lambda_{p}\right)}^{2},$$ (19)

in which

$$P_{y}=F_{y}A_{s}+0.7f_{c}^{\prime }A_{c}.$$ (20)

If *λ*_*r*_ < *λ* (slender section),

$$P_{no}=F_{cr}A_{s}+0.7f_{c}^{\prime }A_{c},$$ (21)

in which

$$F_{cr}=\frac{9E_{s}}{{\left(b/t\right)}^{2}}.$$ (22)

The nominal compressive strengths of the two rectangular infilled specimens such as ST_7t_C24_NO and ITU_4t_C24_O can be computed as given in Tables 6 and 7.

**Table 6:** Nominal compressive strengths without length effects of the two infilled specimens.

Specimen	*λ*	*λ*_*p*_	*λ*_*r*_	*P*_*no*_ (kN)	Section type
ST_7t_C24_NO	50.1	48.0	63.7	6,538.2	Noncompact
ITU_4t_C24_O	43.0	49.9	66.3	7,309.1	Compact

$\lambda_{p}=2.26\sqrt{E/F_{y}}$, $\lambda_{r}=3.00\sqrt{E/F_{y}}$, *P*_*no*_ = nominal compressive strength without consideration of length effects.

**Table 7:** Nominal compressive strengths of the two infilled specimens.

Specimen	*P*_*no*_ (kN)	*P*_*e*_ (kN)	$\frac{P_{no}}{P_{e}}$	*P*_*n*_ (kN)
ST_7t_C24_NO	7,916.1	1,639,093.1	4.830×10^−3^	7,900.1
ITU_4t_C24_O	7,311.5	1,345,260.8	5.435×10^−3^	7,294.9

*P*_*e*_ = elastic buckling strength, *P*_*n*_ = nominal compressive strength with consideration of length effects.

Table 6 indicates that the width-thickness ratio (*λ*) of specimen ST_7t_C24_NO is in the range of noncompact section (*λ*_*p*_ < *λ* ≤ *λ*_*r*_), while that of specimen ITU_4t_C24_O in the range of compact section (*λ* ≤ *λ*_*p*_). Both of the two infilled specimens have much higher the elastic buckling strength (*P*_*e*_) than the nominal strength without consideration of length effects (*P*_*no*_). Therefore, their nominal compressive strengths (*P*_*n*_) are the values very close to *P*_*no*_, as shown in Table 7.

## 4. Test results and discussion

In this section, the detailed test results such as the load-displacement curves and failure modes of the test specimens are discussed and compared with the predictions by LRFD specification discussed in Section 3. The effectiveness of the newly proposed CFT-ITU column system is evaluated in terms of its ductility, initial stiffness and peak strength.

### 4.1 Load-displacement curves and failure modes of the test specimens

**Fig 9:**
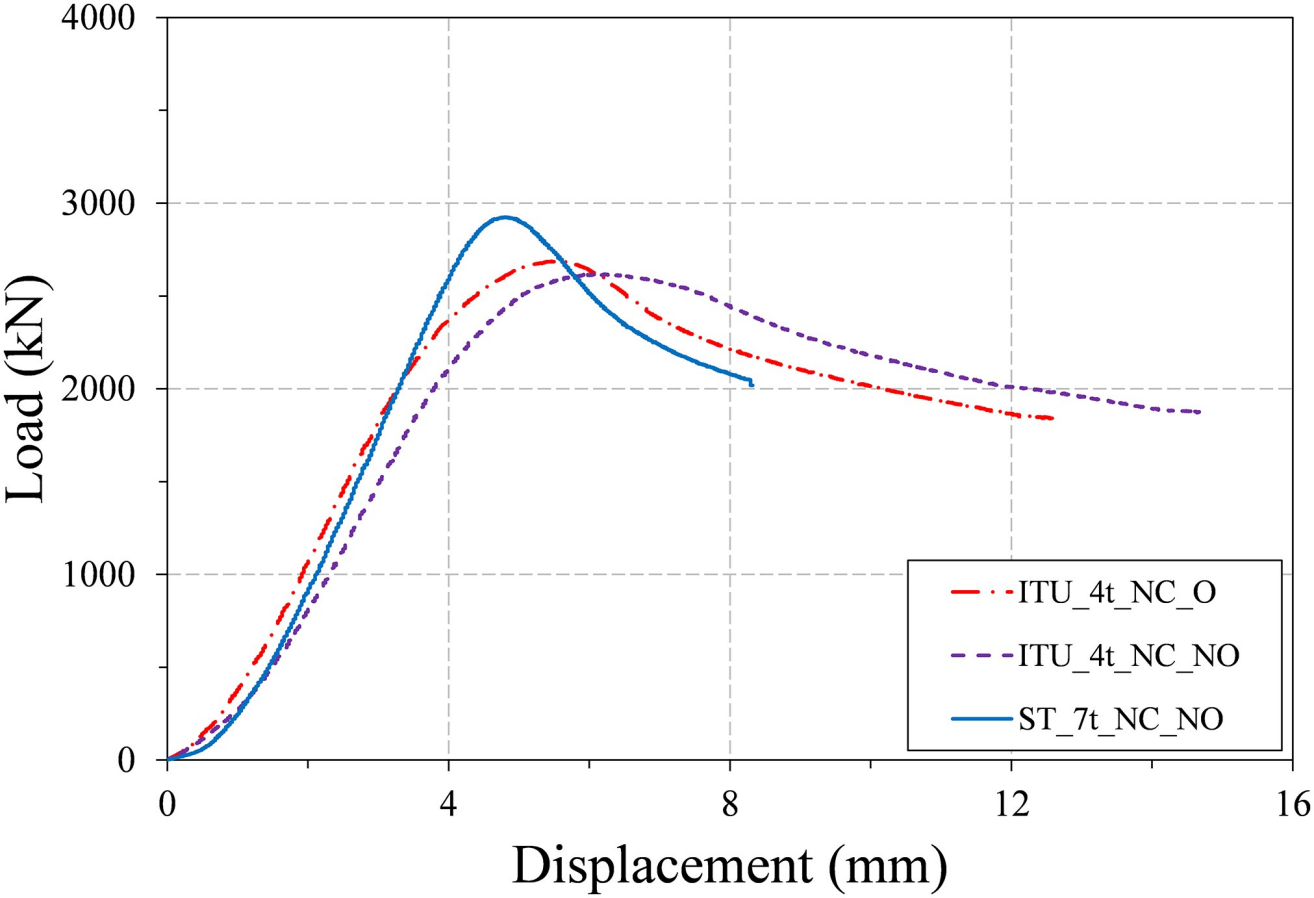
Load-displacement curves of the three non-infilled specimens.

**Fig 10:**
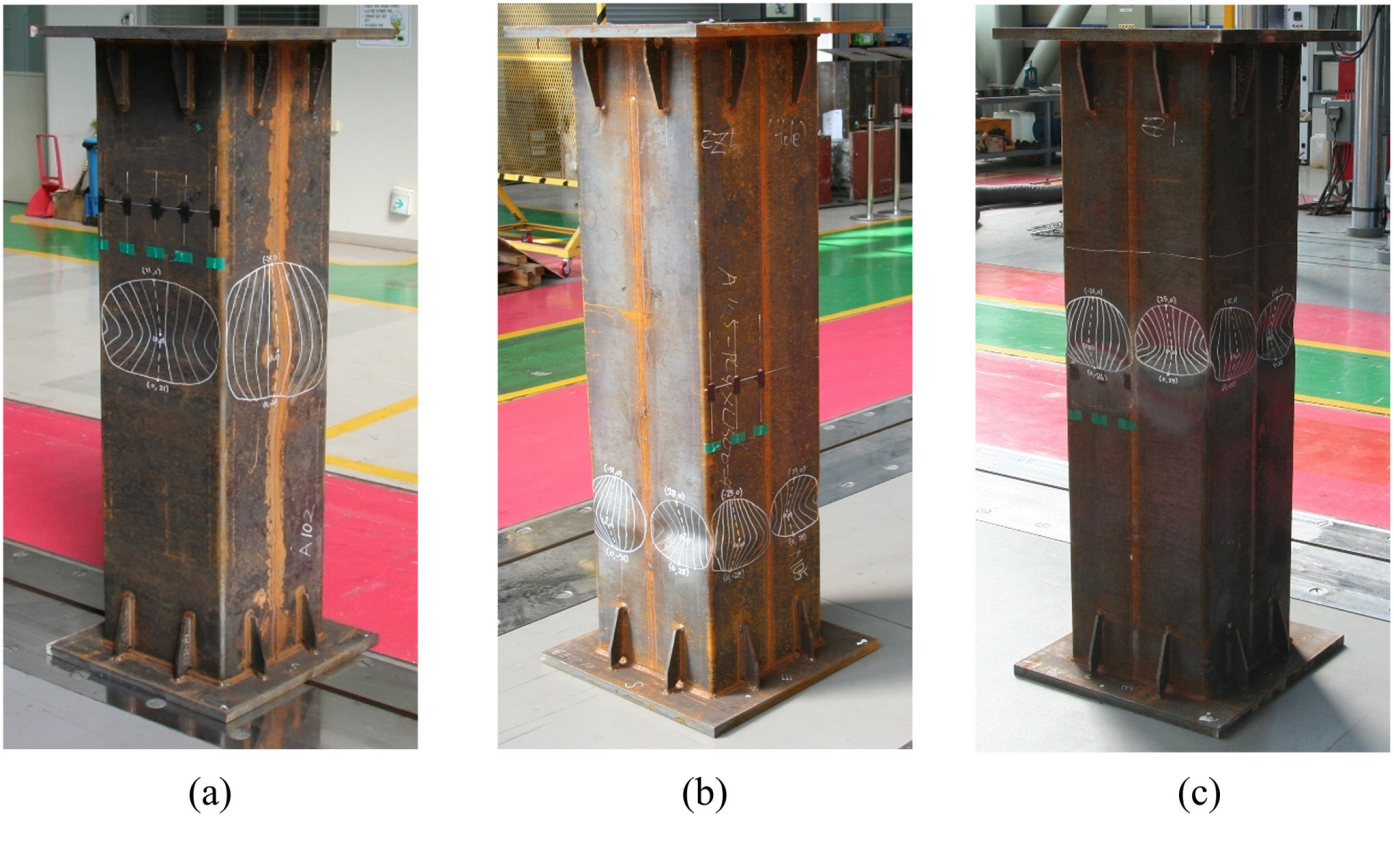
Failure shapes of the three non-infilled specimens. (a) ST_7t_NC_NO. (b) ITU_4t_NC_NO. (c) ITU_4t_NC_O.

The load-displacement curves of the three non-infilled specimens are plotted in Fig 9, and the photos of these specimens taken after the test are provided in Fig 10. All of the three non-infilled specimens showed similar behavior during the test. Their load-displacement curves showed mildly ductile behavior, and load was gradually decreased after reaching the peak strength. The post-peak decrease is slightly more pronounced in the case of the conventional steel tube specimen (ST_7t_C24_NO) than in the other two specimens, but the difference is not so significant.

**Fig 11:**
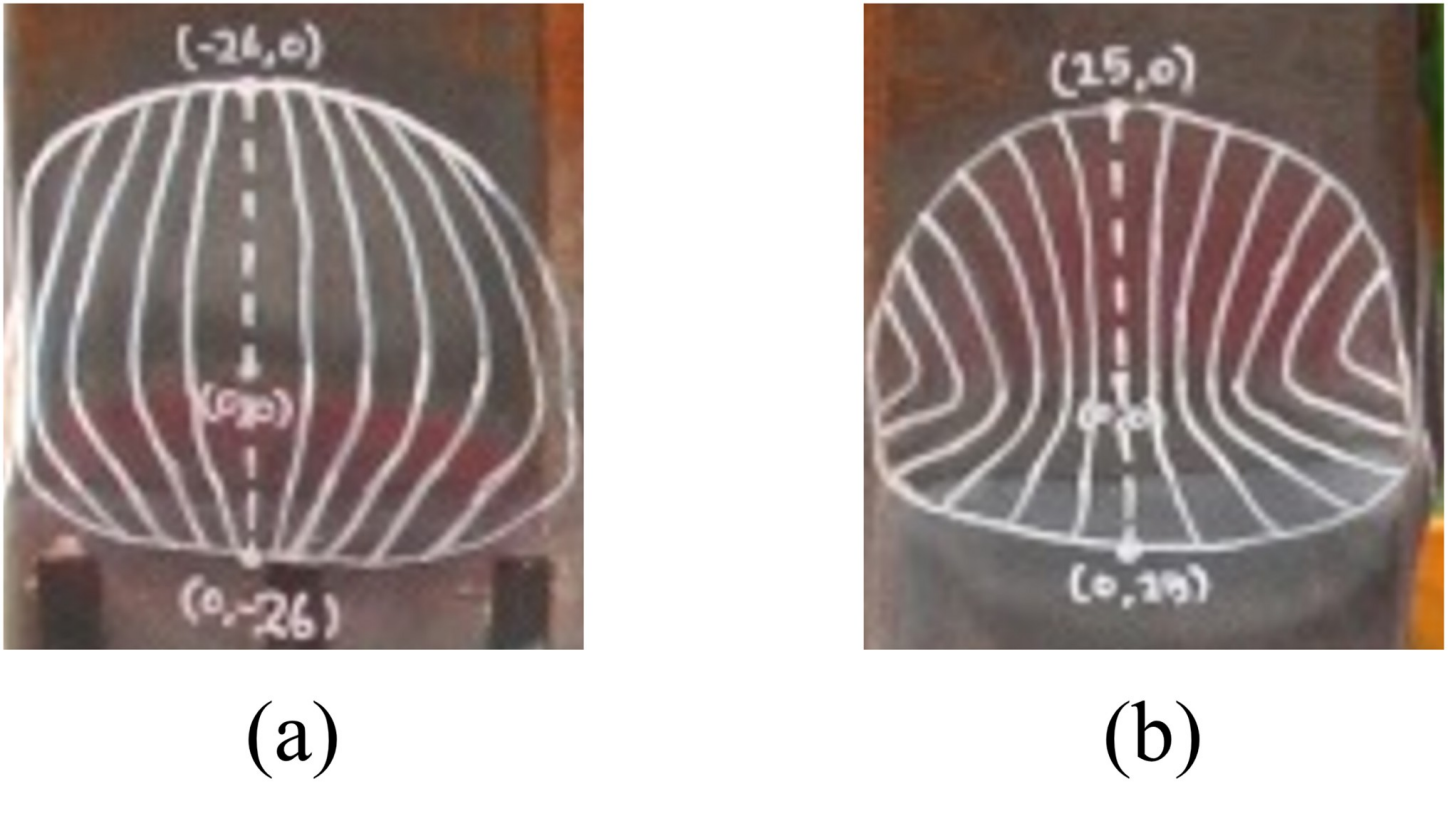
Two types of local buckling shape. (a) Outward direction. (b) Inward direction.

The main failure mechanism observed in these columns is the yielding of the steel tube, attributable to their small effective slenderness ratio, as indicated in Table 5. Moreover, local buckling of the steel tube was noted in all three non-infilled column specimens, as illustrated in Fig 10(a) to 10(c). This observation aligns with the LRFD predictions presented in Table 4, which identify all three non-infilled specimens as having slender cross-sections. The specimens exhibited both outward and inward buckling in the steel tube, illustrated in two distinct manners in Fig 11(a) and 11(b), respectively. This finding is consistent with the observations reported by Han et al. [40].

The peak load values, displacements at peak load and nominal compressive strengths by LRFD specification for the three non-infilled specimens are summarized in Table 8. The ratios of the peak strength (A) and LRFD strength estimation (B) are also provided in the table to validate the accuracy of the LRFD specification. The results of the table indicate that the LRFD compressive strength estimations are generally in good agreement with the test peak strengths except specimen ITU_4t_NC_NO. This seems to happen because there was a small defect in the welding of internal triangular units of this specimen, resulting in decrease in its initial stiffness and peak strength.

**Table 8:** Comparison between the test results and LRFD estimations of the three infilled specimens.

Specimen	Peak strength^(A)^ (kN)	Displacement at peak strength (mm)	Nominal strength by LRFD^(B)^ (kN)	Ratio of (A) to (B)
ST_7t_NC_NO	2,924.5	4.8	2,981.8	0.98
ITU_4t_NC_NO	2,617.5	6.0	3,115.5	0.84
ITU_4t_NC_O	2,687.1	5.6	2,410.5	1.11

**Fig 12:**
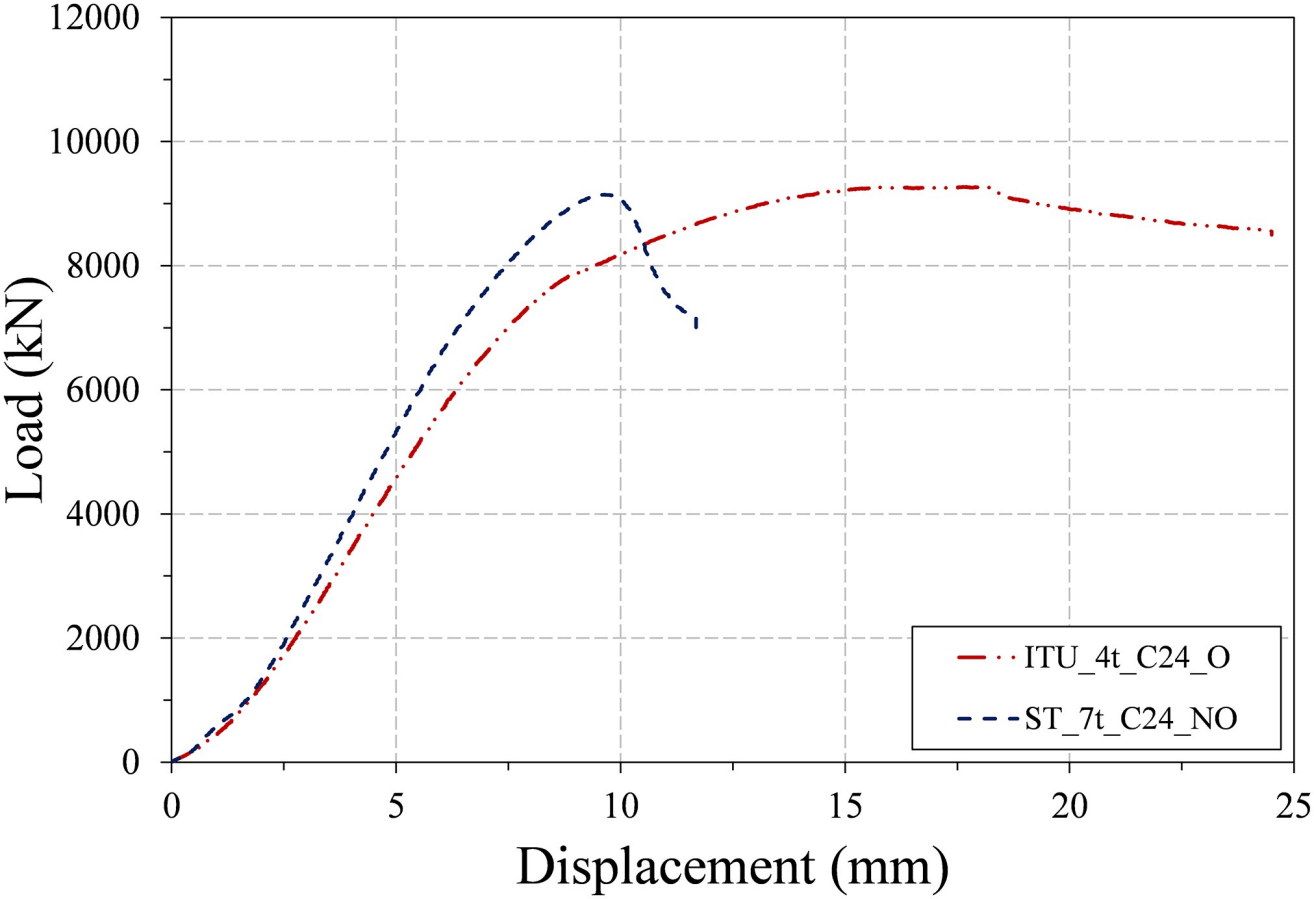
Load-displacement curves of the two infilled specimens.

**Fig 13:**
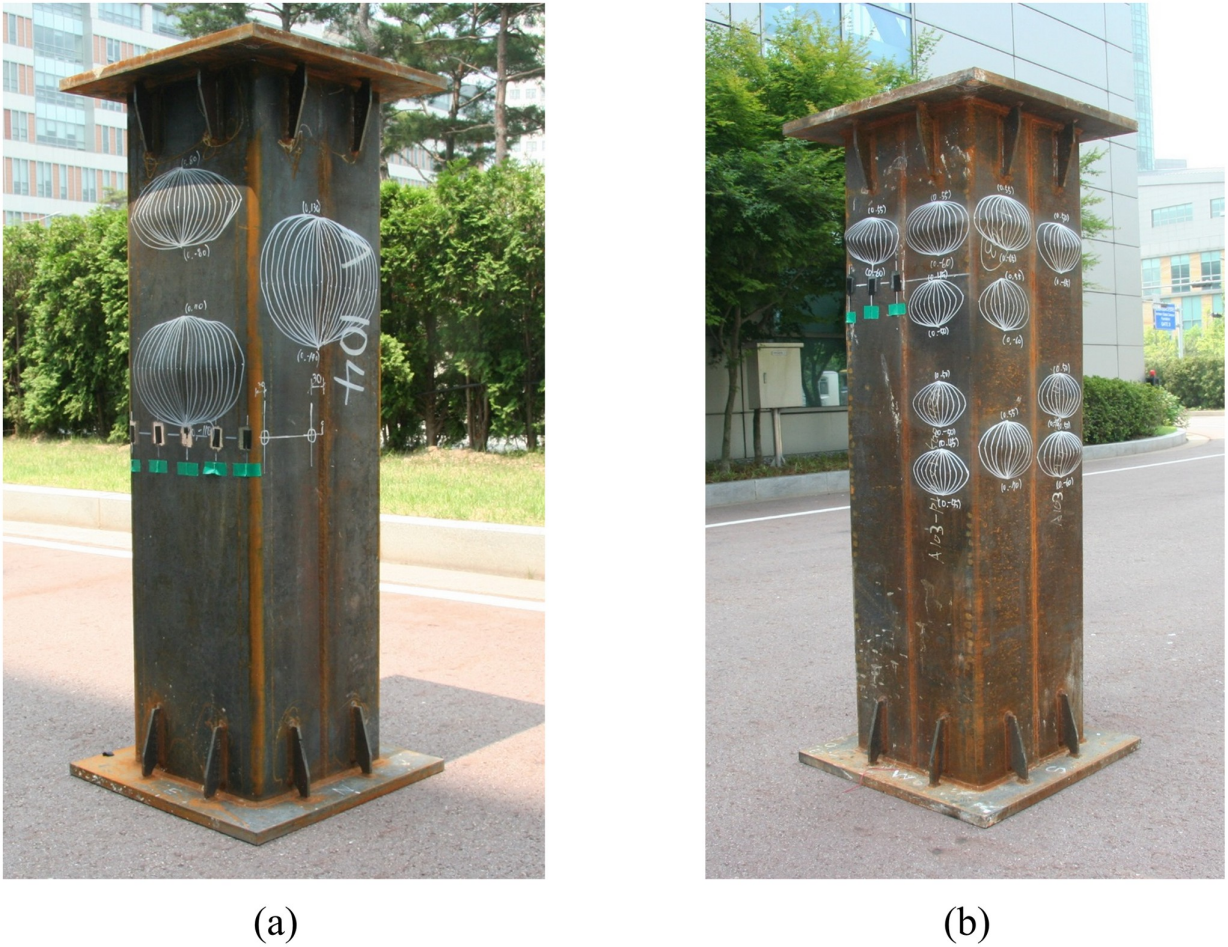
Failure shape of the two infilled specimens. (a) ST_7t_C24_NO. (b) ITU_4t_C24_O.

Fig 12 plots the load-displacement curves of the two infilled specimens, and the failure shapes of these specimens are presented in Fig 13. The results in Fig 12 indicate that the difference between the load-displacement curves of the infilled ST and ITU specimens is significant, in contrast to those of the three non-infilled specimens shown in Fig 9.

The load-displacement curve of the infilled ST specimen (ST_7t_C24_NO) showed rather brittle behavior, and its main failure mechanism is the yielding of steel tubes and crushing of concrete core. Local buckling was also observed in the steel tube and contributed to reducing the slope of the load-displacement curve before reaching the peak strength. In contrast, the load-displacement curve of the infilled ITU specimen (ITU_4t_C24_O) showed more ductile behavior. At the applied load of approximately 7,000 kN, local buckling started to be observed in the steel tube. Then the slope in the load-displacement curve gradually decreased, and the range of local buckling was expanded. Load was gradually decreased after reaching the peak load, resulting in smooth deformation transition from pre- to post-peak.

As depicted in Fig 13, local buckling occurred in both specimens, with only outward buckling observed in the presence of infilled concrete, consistent with findings reported in the work of Wang et al. [41]. Notably, the size of local buckling observed in the infilled internal triangular unit (ITU) specimen is smaller compared to that of the infilled steel tube (ST) specimen. However, the number of instances of local buckling in the infilled ITU specimen is greater than that in the infilled ST specimen. This suggests that the occurrence of small-sized local buckling exclusively in the outward direction can enhance the local stability of concrete-filled tube (CFT) columns, ultimately contributing to an improvement in overall ductility. Further, a detailed discussion on the ductility of each specimen will be provided in Section 4.2.

As similar to Table 8, several parameters such as the peak load values, displacements at peak load and LRFD nominal strengths and the ratios of the test and theoretical strengths are listed for the two infilled specimens in Table 9. They show that the LRFD compressive strength estimations slightly underestimate the test peak strengths, indicating that the LRFD strength equation is a rather conservative estimate especially for the infilled ITU specimen. This seems to happen because the beneficial confinement effect is not considered in the design of the rectangular CFT column, as discussed in Section 1.

**Table 9:** Comparison between the test results and LRFD estimations.

Specimen	Peak strength^(A)^ (kN)	Displacement at peak strength (mm)	Nominal strength by LRFD^(B)^ (kN)	Ratio of (A) to (B)
ST_7t_C24_NO	9,147.0	9.7	7,900.1	1.16
ITU_4t_C24_O	9,271.5	17.6	7,294.9	1.27

### 4.2 Displacement ductility, initial stiffness and peak strength

In order to evaluate the ductility of the five test specimens, the definition of the displacement ductility introduced by Cohn and Bartlett [42] is applied. They proposed a displacement ductility index (*μ*) that is defined as the ratio of the ultimate displacement (*Δ*_*u*_) to the first yield displacement (*Δ*_*y*_).


$$\mu =\frac{\Delta_{u}}{\Delta_{y}}.$$ (23)

**Fig 14:**
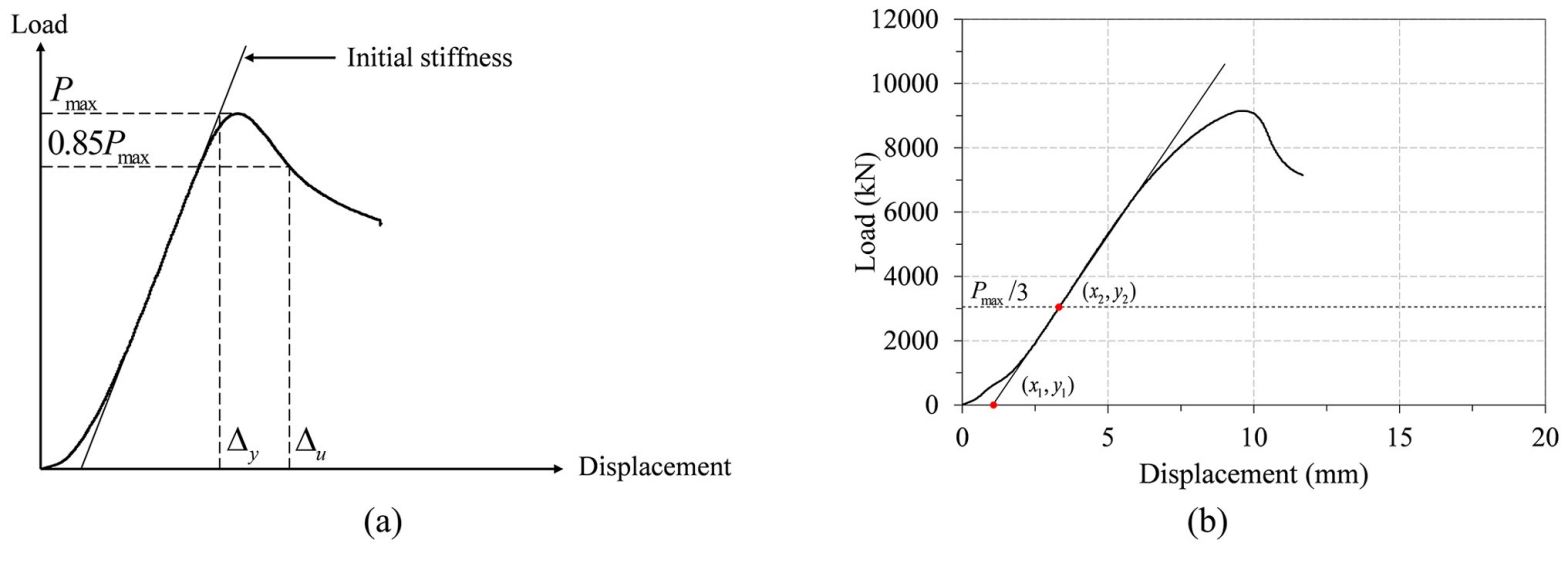
Determination of displacement ductility and initial stiffness. (a) Displacement ductility. (b) Initial stiffness.

As illustrated in Fig 14(a), *Δ*_*u*_ is the displacement corresponding to 85% of the maximum load on the post-peak portion of the curve, and *Δ*_*y*_ is the displacement on the initial stiffness line, of which load value corresponds to the peak load *P*_*max*_. Here, the initial stiffness line is defined as the line, which connects the modified coordinate origin and the point corresponding to one-third of *P*_*max*_ on the load-displacement curve. In all of the test specimens, a small amount of slip occurred at the beginning of the test. Thus, this portion is excluded in the determination of the initial stiffness line as shown in Fig 14(b), as it does not represent any meaningful structural behavior of the test specimen.

**Fig 15:**
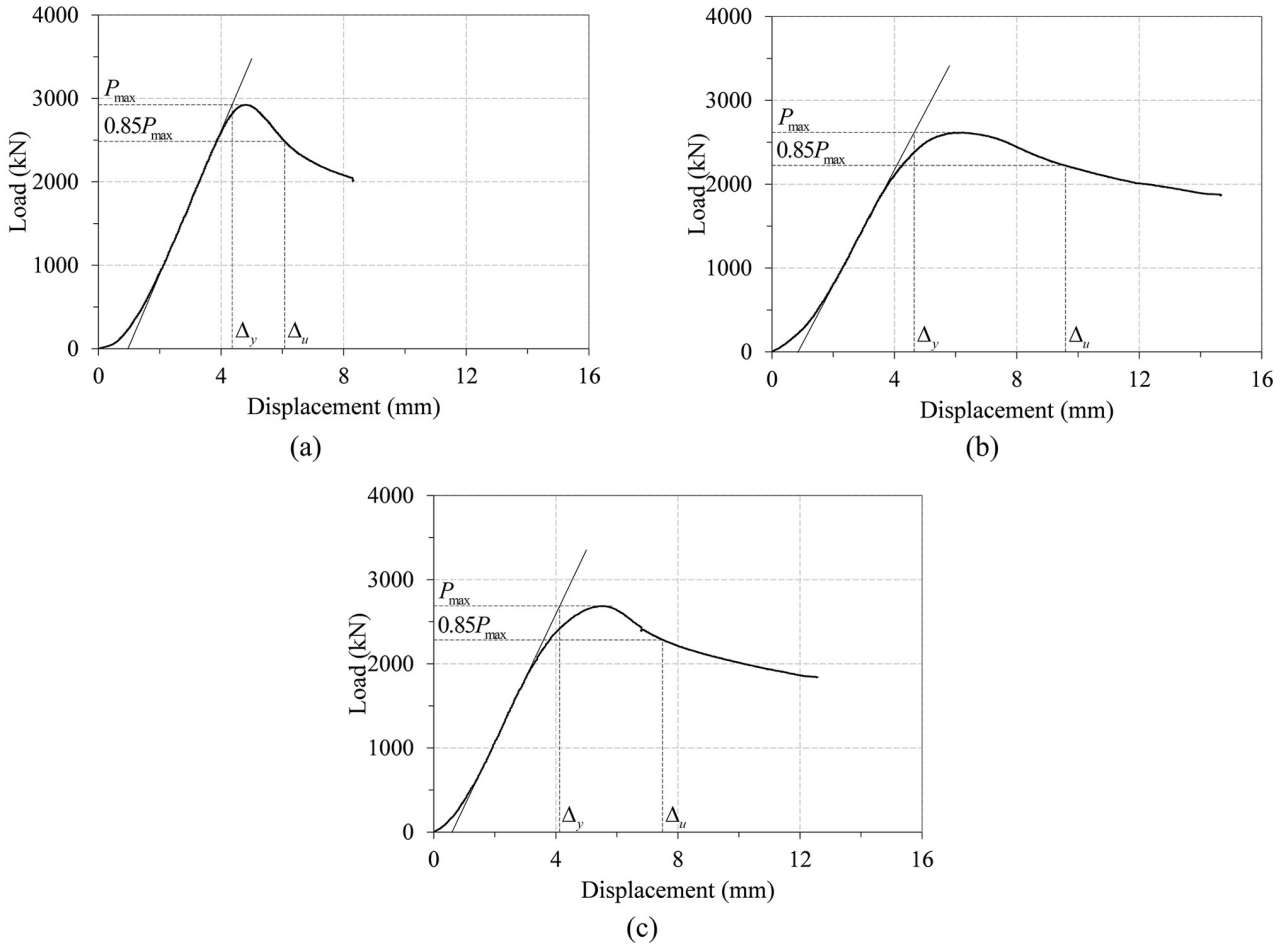
Displacement ductility determination of the three non-infilled specimens. (a) ST_7t_NC_NO. (b) ITU_4t_NC_NO. (c) ITU_4t_NC_O.

**Fig 16:**
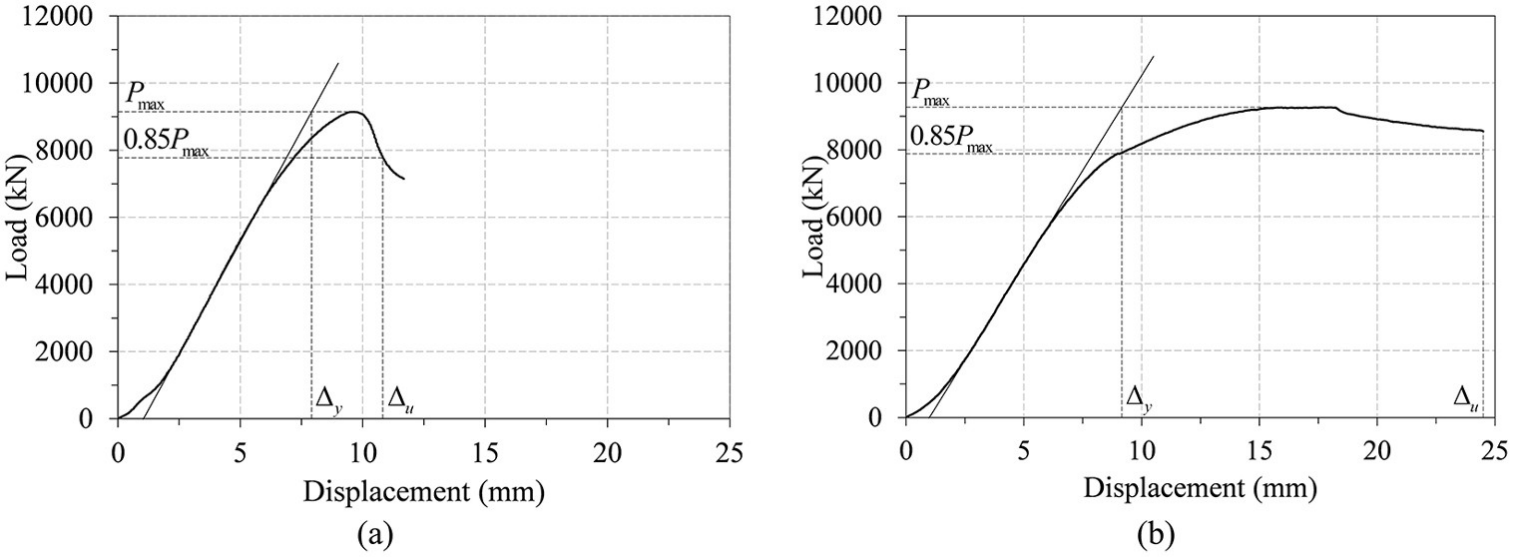
Displacement ductility determination of the two infilled specimens. (a) ST_7t_C24_NO. (b) ITU_4t_C24_O.

Figs 15 and 16 show the determination of the first yield and ultimate displacements for the three non-infilled specimens and two infilled specimens, respectively. They confirm that the displacement ductility index defined by Eq (23) can be reasonably determined for all of the test specimens except specimen ITU_4t_C24_O, in which the test was terminated before reaching the point corresponding to 0.85 *P*_*max*_, as shown in Fig 16(b). Consequently, in this case, *Δ*_*u*_ is replaced by the largest displacement on the load-displacement curve.

Tables 10 and 11 summarize the displacement ductility index results for the three non-infilled specimens and the two infilled specimens, respectively. Among the parameters included in the two tables, the relative displacement ductility index is defined as the ratio of the displacement ductility index of each specimen to that of the conventional steel tube specimen. In the results of Table 10, the two ITU specimens (ITU_4t_NC_NO and ITU_4t_NC_O) have the displacement ductility approximately 30% to 50% higher than that of the conventional steel tube specimen (ST_7t_NC_NO). This difference becomes even greater in the case of the two infilled specimens. The results of Table 11 show that the displacement ductility index of the ITU specimen (ITU_4t_C24_O) is 95% higher than that of the conventional steel tube specimen (ST_7t_C24_NO). This confirms that the newly proposed CFT-ITU column retains much higher ductility than the conventional steel tube column, which is a great advantage in the design of earthquake resistant structures.

**Table 10:** Displacement ductility index for the three non-infilled specimens.

Specimen	Initial stiffness (kN/mm)	Δ_*y*_ (mm)	Δ_*u*_ (mm)	Displacement ductility index	Relative displacement ductility index
ST_7t_NC_NO	860.63	4.36	6.07	1.39	1.00
ITU_4t_NC_NO	689.78	4.64	9.59	2.07	1.49
ITU_4t_NC_O	761.14	4.13	7.5	1.82	1.31

**Table 11:** Displacement ductility index for the two infilled specimens.

Specimen	Initial stiffness (kN/mm)	Δ_*y*_ (mm)	Δ_*u*_ (mm)	Displacement ductility index	Relative displacement ductility index
ST_7t_C24_NO	1332.03	7.91	10.82	1.37	1.00
ITU_4t_C24_O	1134.75	9.16	24.5	2.67	1.95

Table 12 lists the relative values of initial stiffness (B) and steel cross-sectional area (A) with respect to the conventional steel tube specimen for each of the three non-infilled specimens. Here, the steel cross-sectional area of the specimen is proportional to the amount of materials used because all of the specimens have the same height. As a result, the ratio of (B) to (A), denoted as efficiency ratio of initial stiffness, represents the level of initial stiffness that a given specimen can have for the same amount of materials used. The results of the table indicate that this ratio of specimen ITU_4t_NC_O is 16% higher than that of specimen ST_7t_NC_NO, confirming that the newly proposed CFT-ITU specimen retains initial stiffness comparable to or even higher that of the conventional steel tube specimen. As already discussed in Section 4.1, there was a defect in the welding of specimen ITU_4t_NC_NO, leading to decrease in its initial stiffness and peak load.

**Table 12:** Efficiency ratios of initial stiffness for the three non-infilled specimens.

Specimen	*A*_*s*_ (mm^2^)	Relative steel cross-sectional area (A)	Initial stiffness (kN/mm)	Relative initial stiffness (B)	Efficiency ratio of initial stiffness ((B)/(A))
ST_7t_NC_NO	10,752	1.000	860.6	1.000	1.000
ITU_4t_NC_NO	10,602	0.986	689.8	0.801	0.812
ITU_4t_NC_O	8,202	0.763	761.1	0.884	1.159

Note: The values of *A*_*s*_ and the initial stiffness are sourced from Tables 3 and 10, respectively.

Table 13 provides the relative values of peak strength (C) and steel cross-sectional area (A) with respect to the conventional steel tube specimen for each of the three non-infilled specimens. The ratio of (C) to (A), denoted as efficiency ratio of peak strength, represents the level of peak strength that a given specimen can have for the same amount of materials used. The results of the table show that this ratio of specimen ITU_4t_NC_O is 20% higher than that of specimen ST_7t_NC_NO, confirming that the newly proposed CFT-ITU specimen retains peak strength comparable to or even higher that of the conventional steel tube specimen.

**Table 13:** Efficiency ratios of peak strength for the three non-infilled specimens.

Specimen	*A*_*s*_ (mm^2^)	Relative steel cross-sectional area (A)	Peak strength (kN)	Relative peak strength (C)	Efficiency ratio of peak strength ((C)/(A))
ST_7t_NC_NO	10,752	1.000	2,924.5	1.000	1.000
ITU_4t_NC_NO	10,602	0.986	2,617.5	0.895	0.908
ITU_4t_NC_O	8,202	0.763	2,687.1	0.919	1.204

Note: The values of the peak strength are sourced from Table 8.

As similar to Tables 12–15 provide the efficiency ratios of initial stiffness and peak strength for the two infilled specimens, respectively. The results of the tables show that the infilled ITU specimen (ITU_4t_C24_O) has the efficiency ratio of initial stiffness approximately 12% higher than that of the infilled ST specimen (ST_7t_C24_NO). Similarly, the former has the efficiency ratio of peak strength approximately 33% higher than that of the latter. These results confirm that the newly proposed CFT-ITU column system retains both of initial stiffness and peak strength comparable to or even higher those of the conventional steel tube column system.

**Table 14:** Efficiency ratios of initial stiffness for the two infilled specimens.

Specimen	*A*_*s*_ (mm^2^)	Relative steel cross-sectional area (A)	Initial stiffness (kN/mm)	Relative initial stiffness (B)	Efficiency ratio of initial stiffness ((B)/(A))
ST_7t_C24_NO	10,752	1.000	1332.3	1.000	1.000
ITU_4t_C24_O	8,202	0.763	1134.8	0.852	1.117

Note: The values of *A*_*s*_ and the initial stiffness are sourced from Tables 3 and 11, respectively.

**Table 15:** Efficiency ratios of peak strength for the two infilled specimens.

Specimen	*A*_*s*_ (mm^2^)	Relative steel cross-sectional area (A)	Peak strength (kN)	Relative peak strength (C)	Efficiency ratio of peak strength ((C)/(A))
ST_7t_C24_NO	10,752	1.000	9,147.0	1.000	1.000
ITU_4t_C24_O	8,202	0.763	9,271.5	1.014	1.329

Note: The values of the peak strength are sourced from Table 9.

## 5. Conclusions

In this paper, we proposed a new concrete-filled tube column, in which the steel tube consists of four internal triangular units. The presence of the internal triangular units can reduce the width-thickness ratio of the steel tube and increase the effective confinement area of the infilled concrete, leading to enhanced performance and sustainability in terms of structural strength and ductility. A full-scale test was performed on five square steel tube column specimens subjected to axial compression to investigate the effectiveness of the proposed column system. Two of them were the conventional steel tube columns, and the other three specimens were the newly proposed CFT columns with internal triangular units. The shape of the CFT column, the presence of infilled concrete and the presence of openings on the ITUs were considered as test parameters. The main conclusions of this paper are as follows:

1. The load-displacement curves of the three non-infilled specimens showed mildly ductile behavior, and their LRFD compressive strength estimations are generally in good agreement with the test peak strengths. Local buckling of the steel tube in both outward and inward directions was observed for all three non-infilled column specimens.
2. The load-displacement curve of the infilled ST specimen exhibited a relatively brittle behavior, whereas the infilled ITU specimen displayed a more ductile response. The LRFD compressive strength estimations slightly underestimated the test peak strengths. This suggests that the LRFD strength equation is a rather conservative estimate, particularly for the infilled ITU specimen.
3. The main failure mechanism of the two infilled specimens is the yielding of steel tubes and crushing of concrete core. Local buckling only the outward buckling was observed in both specimens due to the presence of infilled concrete. Interestingly, the size of local buckling formed in the infilled ITU specimen is smaller than that of the infilled ST specimen, but its number is larger than that of the infilled ST specimen.
4. The results regarding the displacement ductility of the test specimens demonstrate that the newly proposed CFT-ITU column system maintains significantly higher ductility compared to the conventional CFT column. This is a significant advantage when designing earthquake-resistant structures.
5. The results concerning the initial stiffness and peak strength of the test specimens indicate that the newly proposed CFT-ITU column system retains initial stiffness and peak strength levels that are comparable to, and in some cases even higher than, those of the conventional CFT column.

Currently, we are investigating the lateral resistance of the newly proposed CFT-ITU column system by performing a full-scale test on several test specimens subjected to cyclic lateral loading.

## References

[1] El-Tawil S, Deierlein GG. Strength and ductility of concrete encased composite columns. J. Struct. Eng.-ASCE. 1999; 125: 1009–1019.

[2] Sakino K, Nakahara H, Morino S. Nishiyama I. Behavior of centrally loaded concrete-filled steel-tube short columns. J. Struct. Eng.-ASCE. 2004; 130: 180–188.

[3] Wei Y, Jiang C, Wu YF. Confinement effectiveness of circular concrete-filled steel tubular columns under axial compression. J. Constr. Steel Res. 2019; 158: 15–27.

[4] Sarir P, Jiang H, Asteris PG, Formisano A, Armaghani DJ. Iterative finite element analysis of concrete-filled steel tube columns subjected to axial compression. Buildings. 2022; 12: 2071.

[5] Zhao H, Han R, Yuan W, Zhao S, Sun Y. Elastoplastic Analysis of Circular Steel Tube of CFT Stub Columns under Axial Compression. Materials. 2022; 15: 8275. doi: 10.3390/ma15228275 36431760 PMC9694866

[6] Kwon YB, Seo SJ, Kang DW. Prediction of the squash loads of concrete-filled tubular section columns with local buckling. Thin Wall. Struct. 2011; 49: 85–93.

[7] Park KD, Kim HJ, Hwang WS. Experimental and numerical studies on the confined effect of steel composite circular columns subjected to axial load. Int. J. Steel Struct. 2012; 12: 253–265.

[8] Qin Y, Lu JY, Cao S. Theoretical Study on Local Buckling of Steel Plate in Concrete-filled Tube Column under Axial Compression. ISIJ Int. 2017; 57: 1645–1651.

[9] Chen P, Wang Y, Zhang S. Size effect prediction on axial compression strength of circular CFST columns. J. Constr. Steel Res. 2020; 172: 106221.

[10] Cakiroglu C, Islam K, Bekdaş G, Isikdag U, Mangalathu S. Explainable machine learning models for predicting the axial compression capacity of concrete filled steel tubular columns. Constr. Build. Mater. 2022; 356: 129227.

[11] Wu HP, Qiao GP, Han XD, Ma HX, Cao WL, Xia DR. Compression-bending behavior of irregular pentagonal CFT columns with different cavity structures: Experimental study. J. Build. Eng. 2023; 67: 105972.

[12] Jamaluddin N, Lam D, Dai XH, Ye J. An experimental study on elliptical concrete filled columns under axial compression. J. Constr. Steel Res. 2013; 87: 6–16.

[13] Yang Y, Wang Y, Fu F. Effect of reinforcement stiffeners on square concrete-filled steel tubular columns subjected to axial compressive load. Thin Wall. Struct. 2014; 82: 132–144.

[14] Ekmekyapar T, Al-Eliwi BJ. Experimental behaviour of circular concrete filled steel tube columns and design specifications. Thin Wall. Struct. 2016; 105: 220–230.

[15] Güneyisi EM, Gültekin A, Mermerdaş K. Ultimate capacity prediction of axially loaded CFST short columns. Int. J. Steel Struct. 2016; 16: 99–114.

[16] Shariati M, Naghipour M, Yousofizinsaz G, Toghroli A, Tabarestani NP. Numerical study on the axial compressive behavior of built-up CFT columns considering different welding lines. Steel Compos. Struct. 2020; 34: 377–391.

[17] Ayough P, Wang YH, Zeng W, Hassanein MF, Elchalakani M. Numerical investigation and design of concrete-filled double square steel tube columns under axial compression. J. Constr. Steel Res. 2024; 212: 108277.

[18] Han LH, Yao GH, Zhao XL. Tests and calculations for hollow structural steel stub columns filled with self-consolidating concrete. J. Constr. Steel Res. 2005; 61: 1241–1269.

[19] Lee SH, Choi YH, Kim YH, Choi SM. Structural performance of welded built-up square CFST stub columns. Thin Wall. Struct. 2012; 52: 12–20.

[20] Kwon YB, Jeong IK. Resistance of rectangular concrete-filled tubular (CFT) sections to the axial load and combined axial compression and bending. Thin Wall. Struct. 2014; 79: 178–186.

[21] Zhao YG, Lin S, Lu ZH, Saito T, He L. Loading paths of confined concrete in circular concrete loaded CFT stub columns subjected to axial compression. Eng. Struct. 2018; 156: 21–31.

[22] Lee HJ, Park HG, Choi IR. Eccentric compression behavior of concrete-encased-and-filled steel tube columns with high-strength circular steel tube. Thin Wall. Struct. 2019; 144: 106339.

[23] Naghipour M, Yousofizinsaz G, Shariati M. Experimental study on axial compressive behavior of welded built-up CFT stub columns made by cold-formed sections with different welding lines. Steel Compos. Struct. 2020; 34: 347–359.

[24] American Institute of Steel Construction, Manual of Steel Construction, Load and Resistance Factor Design, 14th ed.; AISC: Chicago, IL, USA, 2010.

[25] BS EN 1994-1-1 Eurocode 4: Design of composite steel and concrete structures, Part 1–1: General rules and rules for buildings; London, UK, 2009.

[26] Machacek J, Studnicka J. Perforated Shear Connectors, Steel Compos. Struct. 2002; 2: 51–66.

[27] Kim YH, Kang JY, Koo HB, Kim DJ. Pull-out resistance capacity of a new perfobond shear connector for steel pile cap strengthening. Adv. Mater. Sci. Eng. 2016; 1374689.

[28] Kim YH, Kang JY, Kim SH, Kim DJ. Structural performance of steel pile caps strengthened with perfobond shear connectors under lateral loading. Appl. Sci. 2016; 6: 317.

[29] Rhim HC, Kang JY, Kim YH, Kim DJ. Push-out test and analysis of steel pile caps strengthened with perfobond shear connectors. Mag. Concrete Res. 2020; 72: 182–193.

[30] Zhou X, Zhou Z, Gan D. Analysis and design of axially loaded square CFST columns with diagonal ribs. J. Constr. Steel Res. 2020; 167: 105848.

[31] Zhang Y, Xu C, Lu X. Experimental study of hysteretic behaviour for concrete-filled square thin-walled steel tubular columns. J. Constr. Steel Res. 2007; 63: 317–325.

[32] Evirgen B, Tuncan A, Taskin K. Structural behavior of concrete filled steel tubular sections (CFT/CFSt) under axial compression. Thin Wall. Struct. 2014; 80: 46–56.

[33] Aslani F, Uy B, Tao Z, Mashiri F. Predicting the axial load capacity of high-strength concrete filled steel tubular columns. Steel Compos. Struct. 2015; 19: 967–993.

[34] Li B, Yang YL, Chen YF, Cheng W, Zhang LB. Behavior of connections between square CFST columns and H-section steel beams. J. Constr. Steel Res. 2018; 145: 10–27.

[35] Ding F, Liu Y, Lyu F, Lu D, Chen J. Cyclic loading tests of stirrup cage confined concrete-filled steel tube columns under high axial pressure. Eng. Struct. 2020; 221: 111048.

[36] Avci-Karatas C. Artificial Neural Network (ANN) Based Prediction of Ultimate Axial Load Capacity of Concrete-Filled Steel Tube Columns (CFSTCs). Int. J. Steel Struct. 2022; 1341–1358.

[37] Chen L, Fakharian P, Eidgahee DR, Haji M, Arab AMA, Nouri Y. Axial compressive strength predictive models for recycled aggregate concrete filled circular steel tube columns using ANN, GEP, and MLR. J. Build. Eng. 2023; 77: 107439.

[38] ASTM Standard C39/C39M-11, Standard test method for compressive strength of cylindrical concrete specimens, Annual Book ASTM Standards: West Conshohocken, PA. 2011.

[39] ASTM Standard A370-12a, 2012. Standard test methods and definitions for mechanical testing of steel products, Annual Book ASTM Standards: West Conshohocken, PA, 2012.

[40] Han LH, Li W, Bjorhovde R. Developments and advanced applications of concrete-filled steel tubular (CFST) structures: Members. J. Constr. Steel Res. 2014; 100: 211–228.

[41] Wang X, Fan F, Lai J. Strength behavior of circular concrete-filled steel tube stub columns under axial compression: A review. Constr. Build. Mater. 2022; 322: 126144.

[42] Cohn MZ, Bartlett M. Computer-simulated flexural tests of partially pre-stressed concrete sections. J. Struct. Div.-ASCE 1982; 108: 2747–2765.

